# Degradation of Sodium Acetate by Catalytic Ozonation Coupled with a Mn-Functionalized Fly Ash: Reaction Parameters and Mechanism

**DOI:** 10.3390/toxics11080700

**Published:** 2023-08-14

**Authors:** Yaoji Chen, Ruifu Chen, Xinglan Chang, Jingying Yan, Yajie Gu, Shuang Xi, Pengfei Sun, Xiaoping Dong

**Affiliations:** 1 Zhejiang Tiandi Environmental Protection Technology Co., Ltd., 2159-1 Yuhangtang Road, Hangzhou 311199, China; samilee2002@sina.com (Y.C.);; 2 Key Laboratory of Surface & Interface Science of Polymer Materials of Zhejiang Province, School of Chemistry and Chemical Engineering, Zhejiang Sci-Tech University, 928 Second Avenue, Xiasha Higher Education Zone, Hangzhou 310018, China

**Keywords:** modified fly ash, ozone catalysis, reaction mechanism, manganese oxide, sodium acetate degradation

## Abstract

Supported ozone catalysts usually take alumina, activated carbon, mesoporous molecular sieve, graphene, etc. as the carrier for loading metal oxide via the impregnation method, sol–gel method and precipitation method. In this work, a Mn-modified fly ash catalyst was synthesized to reduce the consumption and high unit price of traditional catalyst carriers like alumina. As a solid waste discharged from coal-fired power plants fueled by coal, fly ash also has porous spherical fine particles with constant surface area and activity, abd is expected to be applied as the main component in the synthesis of ozone catalyst. After the pretreatment process and modification with MnOx, the obtained Mn-modified fly ash exhibited stronger specific surface area and porosity combined with considerable ozone catalytic performance. We used sodium acetate as the contaminant probe, which is difficult to directly decompose with ozone as the end product of ozone oxidation, to evaluate the performance of this Mn-modified fly. It was found that ozone molecules can be transformed to generate ·OH, ·O_2_^−^ and ^1^O_2_ for the further oxidation of sodium acetate. The oxygen vacancy produced via Mn modification plays a crucial role in the adsorption and excitation of ozone. This work demonstrates that fly ash, as an industrial waste, can be synthesized as a potential industrial catalyst with stable physical and chemical properties, a simple preparation method and low costs.

## 1. Introduction

Fly ash is mainly a solid waste discharged from coal-fired power plants fueled by coal, and the significant accumulation of fly ash in major power plants has posed a threat to the surrounding environment and people’s health [1,2]. While fly ash has been traditionally used in landscaping and the construction of roads, pavements, roadbeds, and embankments [3,4], the development of new methods for the disposal of fly ash is still an urgent need. In recent years, more attention has been paid to environmental functional materials modified by fly ash. For pollutants in water such as organic dyes, antibiotics, heavy metal, nitrogen and phosphorus pollutants, developing efficient adsorption materials, oxidizing catalysts, and new advanced oxidation processes (AOP) technology based on fly ash-modified materials has been widely considered [5,6,7].

The utilization of fly ash as a catalyst for environmental pollutant treatment offers a significant advantage in relation to using waste to treat waste. The physical and chemical properties of the fly ash surface can be thoroughly explored to enhance its modification potential, which can thus replace some expensive traditional catalytic materials. The most common methods for modifying fly ash are acid-base modification, thermal modification, salt modification, and organic modification, according to current research [8,9]. For example, the application of NaOH treatment resulted in the enhanced specific surface area of fly ash, which can further lead to the significant adsorption performance of cadmium in water [10]. The study results show that the direct activation of materials with NaOH solution yielded higher adsorption capabilities compared to untreated fly ash. The maximum adsorption capacities recorded ranged between 9.18 mg/g and 48.5 mg/g [11]. Besides this, the surface oxidation properties of fly ash can also be greatly improved by introducing special metal oxides. For example, CoFe_2_O_4_ can be loaded on fly ash as a nanocomposite and was employed as a highly effective Fenton-like catalyst for the treatment of polymer-flooding wastewater. By optimizing reaction conditions, a notable removal rate of polyacrylamide (70.3%) was achieved [12]. As we all know, the composition of SiO_2_ and Al_2_O_3_ in fly ash accounts for 60–80% [13,14], which also makes the synthesis of zeolite with fly ash as a silicon and aluminum source even more fascinating. Studies have proven that the mesoporous SBA-15 and MCM-41 synthesized with low-cost fly ash as the silicon sources also demonstrate good potential for adsorption/separation applications [15,16].

As one of the AOP technologies, catalytic ozone oxidation technology is widely used in the field of organic wastewater oxidation. The key method is to develop catalysts and reduce the use amount of ozone as far as possible, so as to achieve excellent mineralization for target pollutants. The supported ozone catalysts usually take metal oxide as the active component of the catalyst for loading metal oxide via the impregnation method, sol–gel method and precipitation method [17,18,19]. Like most common AOP technologies used in wastewater treatment processes, the active component is loaded on the surface of the porous carrier [20,21], which endows the catalyst with not only high activity, good stability and easy recovery, but also low costs. In the ozone catalytic oxidation process, pollutants are eliminated by the direct oxidation of ozone in the water, while the oxidation process is induced by the activation of ozone at the active site of the catalyst into reactive oxygen species (ROS) [22].

In this work, we synthesized a MnOx-loaded catalyst using fly ash as a catalyst for the purpose of investigating the ozone-catalyzed oxidation process. As reported in recent studies, replacing precious metals with transition metal oxide (TMO) can greatly decrease the catalyst’s cost [23]. Among a variety of TMOs, manganese oxides are environmentally friendly and cheap, and show plentiful valence and effective light absorption, and so they have become one of the most widely investigated AOP technologies. Sodium acetate, which is difficult to directly decompose using ozone as the end product of ozone oxidation, was selected as the contaminant probe [24]. The obtained modified fly ash catalysts were systematically characterized using various techniques, and their ozone catalytic performance was also evaluated by regulating different reaction parameters. The experimental results reveal that the specific surface and pore structure of the modified fly ash were significantly enhanced, and the content of surface active oxygen species greatly increased after manganese loading. Sodium acetate was eliminated by the simultaneous oxidation of ·OH, ·O^2−^ and ^1^O_2_ generated on the catalyst surface after the addition of ozone.

## 2. Materials and Methods

### 2.1. Pretreatment Process

Fly ash was obtained from the Zhoushan Power Plant of National Energy Group, China (Beijing, China). The fly ash was pretreated as follows: 20 g fly ash and 200 mL 1.2 mol/L HCl solution were stirred at 85 °C for 2 h, and washed with deionized water to neutrality. Subsequently, it was centrifuged and dried at 50 °C. The fly ash sample obtained by acid treatment was named CFA(A).

In total, 20 g CFA(A) and 100 mL 5 mol/L NaOH solution were thermally treated at 90 °C in a hydrothermal kettle for 15 h, washed with deionized water and ethanol, centrifuged to neutrality, and dried at 50 °C. The fly ash sample obtained by acid and alkali treatment was named CFA(A+B).

### 2.2. Preparation of Catalyst

MnOx (at 4%, 8% and 12% in mass ratio) was loaded onto the CFA(A+B) via a common wet impregnation route as reported in the literature [25]. Accurately measured Mn(NO_3_)_2_ and CFA(A+B) were mixed in deionized water, followed by continuous stirring for 5 h. The mixture was then dried at 90 °C for 10 h and calcinated at 400 °C for 5 h in static air. The obtained samples were denoted as Mnx/CFA(A+B), where x was 0.04, 0.08 and 0.12, respectively.

### 2.3. Characterization of Catalyst

The wide-angle X-ray diffraction (XRD) patterns of different fly ash samples were measured by an X-ray powder diffractometer (Haoyuan DX-2700, Dandong, China) with Cu Ka radiation (35 kV, 25 mA). The morphology was observed and recorded with a scanning electron microscope (SEM, Hitachi SU-8100, Tokyo, Japan), and a transmission electron microscope (TEM, JEM 2100, Tokyo, Japan) was also used to further observe the microstructure of the MnOx loaded onto the fly ash samples. Nitrogen adsorption–desorption isotherms were detected by an ASAP 2020 analyzer (Micromeritics, Norcross, GA, USA). Specific surface area and pore size distribution were determined using the Brunauer–Emmett–Teller (BET) and Barrett–Joyner–Halenda (BJH) methods. The sample was degassed at 300 °C for 4 h before the actual measurement. The total pore volume was obtained based on the single-point method at a relative pressure (P/P_0_) of 0.99. X-ray photoelectron spectroscopy (XPS) was measured using a Kratos AXIS Ultra DLD spectrometer (Shimadzu, Kyoto, Japan) equipped with Al Ka X-rays. Data were corrected for a C1s peak of 284.8 eV. Inductively Coupled Plasma Emission Spectra (ICP-OES) were measured using an iCAP PRO XP ICP-OES emission spectrometer (Thermo, Waltham, MA, USA). Electron paramagnetic resonance spectroscopy (EPR) was measured using an A300 EPR spectrometer (Bruker, Karlsruhe, Germany) for detecting vacancies over the catalyst.

### 2.4. Catalytic Ozonation Reaction

Ozone was generated from oxygen with a high purity of 99.9% using a laboratory ozone generator (M-1000, Tonglin, Beijing) with a gas flow rate of 200 mL/min. The ozone was bubbled into a self-designed reactor through a porous titanium aerator with concentration determined by the sodium indigo disulphonate method. A 600 mL solution of sodium acetate (100~250 mg/L) was added to the reactor at a catalyst dosage of 1~4 g/L. The pH was adjusted by adding NaOH or HCl solution with concentration at 0.1 mol/L.

In the catalytic ozonation process, a 4.0 mL sample was taken at certain time intervals, and then filtered using an inorganic filter membrane with a thickness of 0.45 μm. The residual ozone was immediately quenched by adding 0.01 mol/L Na_2_S_2_O_3_ solution. The elimination rate of sodium acetate was determined using a chemical oxygen demand (COD) tester (COD-571, Leici, China).

## 3. Results and Discussion

### 3.1. Characteristics of Pre-Treated Fly Ash Sample

**Figure 1:**
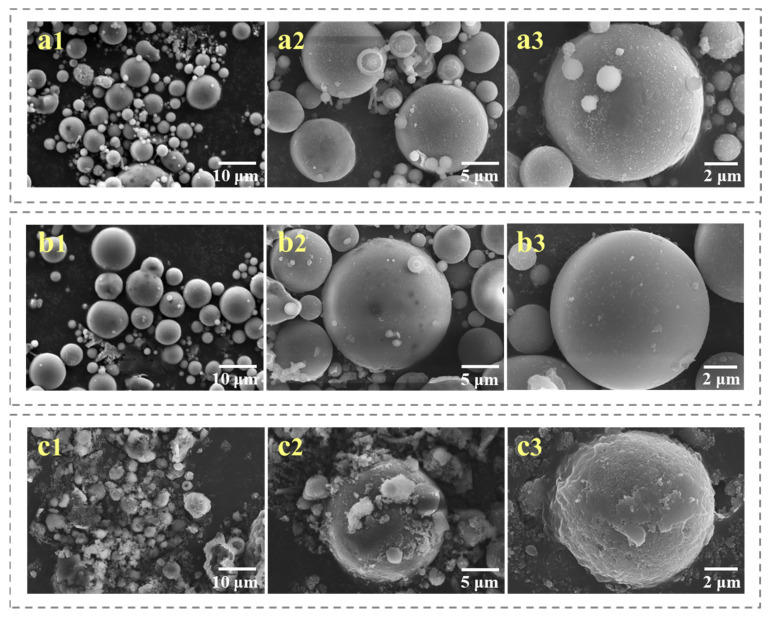
SEM images of (**a1**–**a3**) original CFA, (**b1**–**b3**) CFA(A) and (**c1**–**c3**) CFA(A+B).

**Figure 2:**
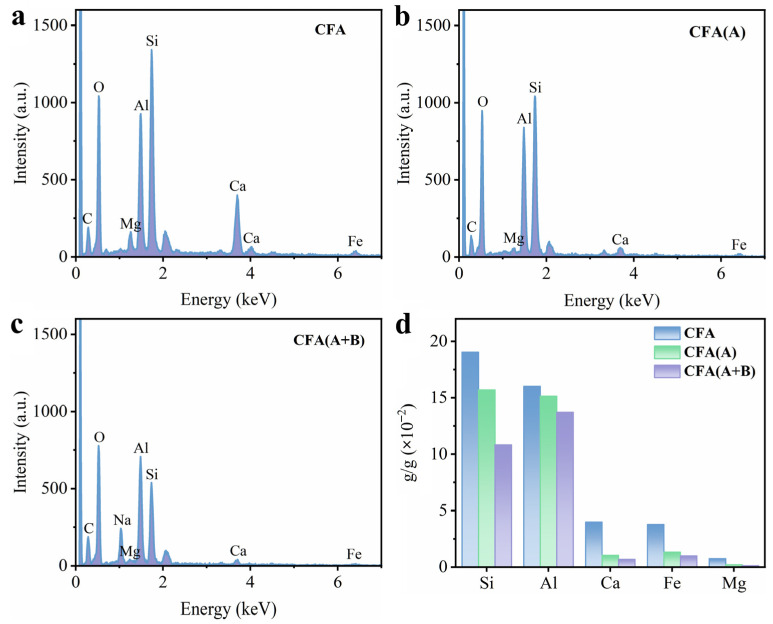
EDS result of (**a**) CFA, (**b**) CFA(A) and (**c**) CFA(A+B); (**d**) ICP analysis of CFA, CFA(A) and CFA(A+B).

The size of specific surface area and pore structure are particularly important for the liquid phase ozone reaction process. As the product of sintering at high temperature, fly ash has a very low specific surface area, so it is not suitable to be used as a catalyst carrier for loading or direct reprocessing. The SEM images do not show obvious surface difference after acid treatment between CFA and CFA(A) samples, as shown in Figure 1. For the CFA(A) sample, the increased S*_BET_* and V*_total_* compared with parent CFA was attributed to the alkali metals and iron ions accumulated on the surface in the formation process dissolved via the acid treatment, along with the release of the pore structure [22]. The EDS and ICP results in Figure 2 and Table 1 also prove that the calcium ions, magnesium ions and iron ions on the surface of the original fly ash were significantly reduced under the action of acid treatment, although there was no obvious surface difference in SEM profiles between CFA and CFA(A). Apparently, pores formed on the surface failed to make the fly ash a better catalyst carrier at this step.

After a further alkali treatment, the sample CFA(A+B) exhibited in Figure 1 had a large crack on the surface, accompanied by reduced particle size, mainly due to the high concentration of alkali treatment, which resulted in the dissolution of a large amount of sodium silicate, thus destroying the original structure of the fly ash surface [26,27,28]. Table 1 compares the S*_BET_* and V*_total_* values of the CFA(A+B) area (59.64 m^2^/g and 0.13 cm^3^/g) with those of the CFA(A) (11.37 m^2^/g and 0.008 cm^3^/g).

### 3.2. Characteristics of Mn-Modified Samples

**Figure 3:**
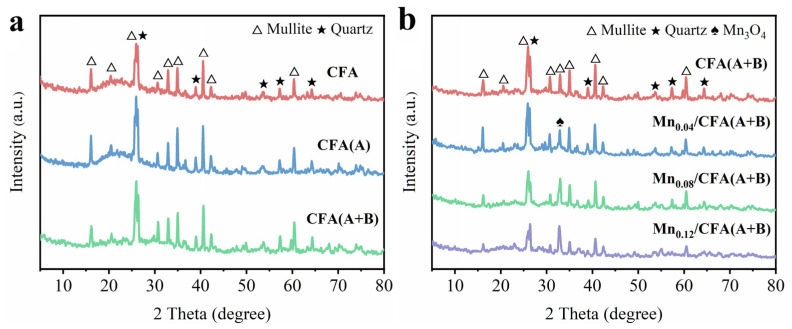
(**a**) The XRD patterns of CFA, CFA(A) and CFA(A+B). (**b**) The XRD patterns of different Mn-modified CFA(A+B).

The XRD pattern of pristine fly ash is shown in Figure 3a, which displays the characteristic peaks of mullite (2 theta at 16.4°, 26.1°, 30.7°, 33.0°, 35.2°, 40.8°, 42.4° and 60.6°) and quartz (2 theta at 20.8°, 25.9° 39.2°, 53.5°, 56.8° and 63.7°) [29]. The diffraction peak of CFA(A) and CFA(A+B) did not change after acid treatment and alkali treatment, indicating that the surface composition was not broken in the pore-making process. According to the XRD pattern in Figure 3b, no obvious diffraction peak of manganese oxide was detected over CFA(A+B) samples with different manganese loading, with only a characteristic peak of Mn_3_O_4_ appearing at 2 theta = 32.4° [30], which was probably caused by highly dispersed manganese oxide due to the small load amount. On the other hand, a calcination temperature of 400 °C was also reported to facilitate the formation of amorphous manganese oxide, which was difficult to identify using XRD [31]. The ICP tests results for Mn loading amount are also listed in Table 2; the actual Mn contents were 3.61%, 6.97% and 10.11% of Mn_0.04_/CFA(A+B), Mn_0.08_/CFA(A+B) and Mn_0.12_/CFA(A+B), respectively, which are close to the theoretical values.

**Figure 4:**
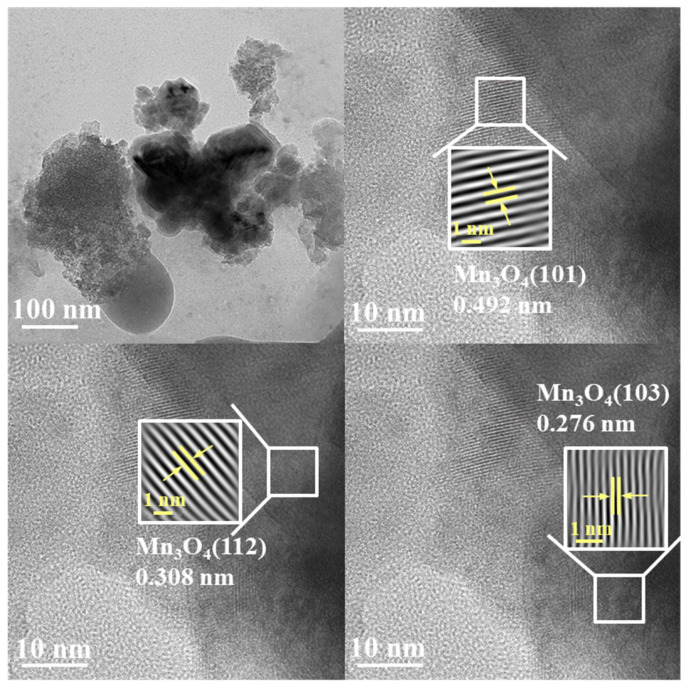
TEM images of synthesized Mn_0.12_/CFA(A+B).

The TEM images of Mn_0.12_/CFA(A+B) are exhibited in Figure 4. It can be seen from the figure that there was no agglomeration of manganese oxide, indicating again its good dispersion over CFA(A+B). The measured lattice spacing of 0.492 nm, 0.308 nm and 0.276 nm was attributed to the (101), (112) and (103) crystal planes of Mn_3_O_4_ (JCPDS card No.18–0803) [32,33,34], indicating the formation of polycrystalline manganese oxide.

Besides this, the SEM images of original CFA, CFA(A), and CFA(A+B) after Mn loading are listed in Figure S1 of the Supplementary Material. The loading of MnOx did not change the morphology values of the original CFA, CFA(A), and CFA(A+B), which were very close to the morphology values listed in Figure 1. It could also be found that the irregular pore structure of fly ash after alkali treatment was obvious, which should be more conducive to the loading of metal oxides.

**Figure 5:**
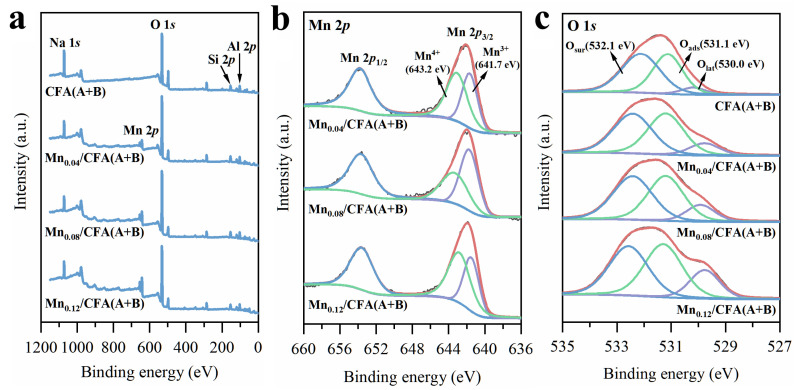
XPS spectra of CFA(A+B) and different Mn-modified CFA(A+B): (**a**) survey; (**b**) Mn 2p; (**c**) O 1s.

To identify the states of surface chemical elements on the different CFA(A+B) samples, XPS analyses were also conducted, and the spectra were deconvoluted by fitting Gaussian peaks after Shirley background subtraction. As shown in Figure 5a, the peaks at the binding energies of 645 eV and 532 eV were ascribed to Mn 2p and O 1s of different CFA(A+B) samples. Obviously, compared with Mnx/CFA(A+B) samples, there was no Mn 2p peak over CFA(A+B). As shown in Figure 5b, the Mn 2p spectra were divided into two main peaks with a binding energy of ca. 653.9 and ca. 642.1 eV, which were respectively assigned to Mn 2p1/2 and Mn 2p3/2 [35]. The peaks at ca. 643.2 eV and 641.7 eV were assigned to Mn^4+^ and Mn^3+^ [36]. According to the peak-splitting results, the Mn^3+^/Mn^4+^ molar ratio of Mn-loaded samples exhibited the maximum value of Mn_0.08_/CFA(A+B), as listed in Table 1. According to the literature [37], the bond energy of Mn(3+)-O is weaker than that of Mn(4+)-O, which makes it easier to release oxygen atoms from the structure with the increase in Mn^3+^/Mn^4+^. Thus, the increased Mn(3+)-O bond will be more involved in the dissociation and activation of surrounding ozone by giving an electron to generate new oxidation groups.

Figure 5c also exhibits the peak-splitting result of O 1s. The BE value in the range of 529.7~530.2 eV was assigned to lattice oxygen (O_lat_) [38,39], and that in the range of 532.1~532.5 eV to surface chemisorbed oxygen or the ionization of oxygen, such as OH^−^, O_2_^−^ and CO_3_^2−^ [40], denoted as O_sur_. The BE peak at 531.1~531.2 eV was ascribed to the oxygen in adsorbed water species [41], which is denoted as O_ads_. Generally, the activation of lattice oxygen requires higher energy. Therefore, this part of O_lat_ cannot easily take part in the oxidation process. However, the surface chemisorbed oxygen (O_sur_) containing different surface reactive oxygen species could easily capture free electrons and act as active sites for ozone adsorption and decomposition into free radicals and non-free radicals, which played a more important role in the ozone catalytic process at room temperature. According to the deconvoluted O 1s spectral data listed in Table 1, the proportion of both O_sur_ and O_lat_ increased with the decrease in O_ads_. The decrease in oxygen in water could be caused by the formation of MnOx occupying parts of the original adsorption sites over the CFA(A+B) sample, which inhibited water adsorption at these sites.

**Figure 6:**
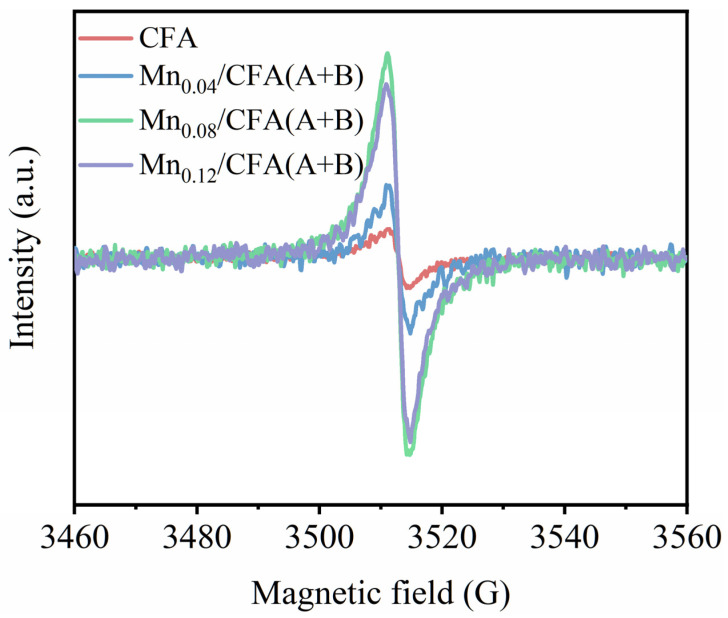
ESR spectra of CFA(A+B) and different Mn-modified CFA(A+B).

The ESR spectra in Figure 6 show that all samples exhibited a pair of steep peaks with a symmetric distribution in accordance with g = 2.002, an indication of electron trapping at oxygen vacancies [42]. Compared with Mn_0.04_/CFA(A+B), the Mn_0.08_/CFA(A+B) exhibited a large increase in oxygen vacancy intensity, which was attributed to the absence of oxygen in the phase and the generation of more Mn^3+^ species (see Table 2, ratio of Mn^3+^/Mn^4+^) to maintain an electric neutrality during calcination. However, the intensity of oxygen vacancy did not further increase over the Mn_0.12_/CFA(A+B) catalyst, and the ratio of superficial Mn^3+^/Mn^4+^ decreased. More Mn(4+)-O was formed in bulk lattice oxygen as the content of manganese was increased to 12 wt.%,which thus resulted in a significant increase in O_lat_ species, but the O_sur_ species did not increase obviously (see Table 2).

### 3.3. Catalytic Ozonation Performance

#### 3.3.1. Effect of Loading Amount and Catalyst Carrier

**Figure 7:**
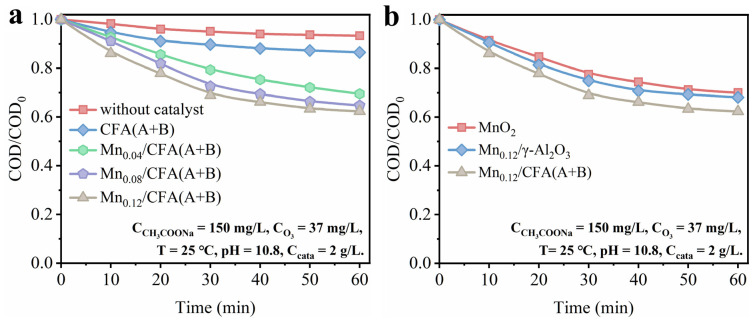
(**a**) The degradation performance of sodium acetate over CFA(A+B) and different Mn-modified CFA(A+B). (**b**) The comparison of MnO_2_, Mn_0.12_/γ-Al_2_O_3_ and Mn_0.12_/CFA(A+B) for sodium acetate removal.

As a small molecule, acetic acid is a typical product at the end of ozone catalytic oxidation, which is hard to directly oxidize using ozone in solution [43]. Thus, sodium acetate was used as the target pollutant in this work to evaluate the ozone catalytic performance of the fly ash-modified catalyst. As shown in Figure 7a, the catalytic ozonation performance of sodium acetate was tested under the condition of an ozone concentration of 37 mg/L, a temperature of 25 °C, a pH of 10.8 and a catalyst amount of 2 g/L. The results show that the removal rate of sodium acetate was significantly increased when MnOx was increased from 4 wt.% to 8 wt.%. From the ESR and ratio of Mn^3+^/Mn^4+^ in the Mn 2p XPS results, we can infer that the ozone molecules could be more easily adsorbed and excited on the surface of Mn_0.08_/CFA(A+B) due to the highest level of oxygen vacancy and Mn(3+)-O. These formed oxygen vacancies and generated electron-rich sites via charge rearrangement in the synthesis process of Mnx/CFA, which acted as an electron donor and was favorable to chemical bonding with ozone and the formation of new ROS for the further oxidation of sodium acetate [44]. However, the performance of the catalyst was also improved as Mn loading was further increased to 12 wt.%, albeit very slightly. This slightly increased removal rate of sodium acetate could have been caused by the direct oxidation of O_lat_. As shown in Table 1, the amount of O_lat_ was increased with the increase in Mn content. According to the literature [17,19], lattice oxygen also shows a certain oxidation performance during the oxidation removal of organic molecules.

In addition, to confirm the importance of the pretreatment of fly ash for improving the surface structure and loading of MnOx, the removal performances of sodium acetate over catalysts Mn_0.08_/CFA(A+B), Mn_0.08_/CFA(A), and Mn_0.08_/CFA were also compared. As shown in Figure S2 of the Supplementary Material, the Mn_0.08_/CFA(A+B) exhibited obvious superiority over Mn_0.08_/CFA and Mn_0.08_/CFA(A). The original mullite and quartz on the surface of fly ash are relatively inert silicon oxides, and a strong alkali could break these structures and provide more effective pores, which is conducive to the loading of manganese oxide and subsequent reactions.

The performances of the Mn_0.12_/CFA(A+B) catalyst, γ-Al_2_O_3_-supported manganese oxide catalyst and pure manganese oxide powder catalyst prepared by the gel–sol method were also compared, as shown in Figure 7b. It can be observed from the figure that the Mn_0.12_/CFA(A+B) catalyst exhibited the best sodium acetate elimination efficiency, indicating that fly ash acting as a catalyst carrier contributed to better performance. According to the ICP analysis exhibited in Figure 2, the pretreated fly ash used as a catalyst carrier still contained a certain proportion of iron oxide. Iron oxide was reported to show a strong ozone oxidation performance, as the superoxide radical (·O_2_^−^) could be produced more efficiently via the electron feeding process between Fe (II) and Fe (III) [45]. Besides this, surface manganese oxide and iron oxide also generated composite oxide by rearranging the metal phases during calcination, which was also beneficial to the oxidation process, resulting in acquiring higher electron mobility [46].

#### 3.3.2. Effect of Different Reaction Parameter

**Figure 8:**
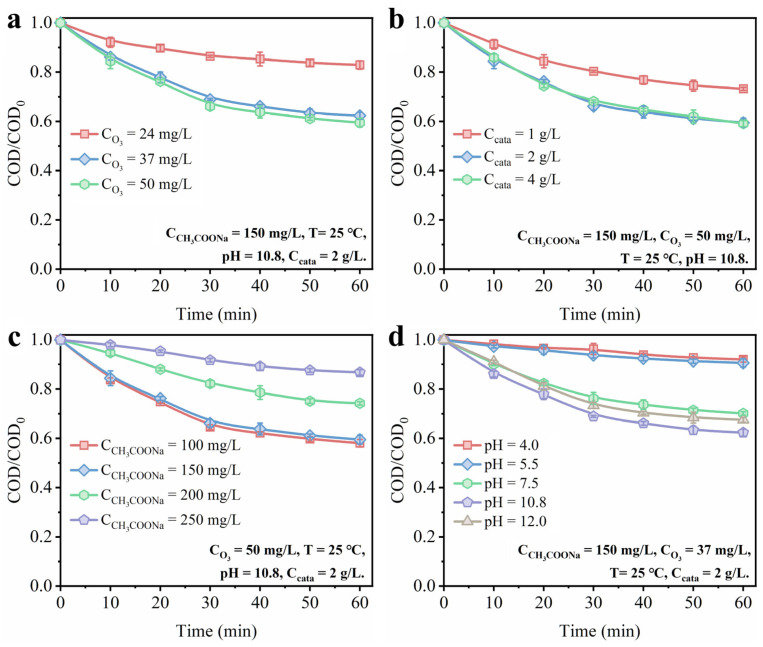
The impact of different reaction parameters on the performance of sodium acetate removal: (**a**) ozone content; (**b**) dosage of catalyst; (**c**) concentration of sodium acetate; (**d**) pH.

As shown in Figure 8a, different ozone concentrations were adopted to evaluate the catalyst performance of the catalyst Mn_0.12_/CFA(A+B). With the increase in ozone concentration from 24 mg/L to 37 mg/L, the removal efficiency of sodium acetate gradually increased. However, the removal efficiency of sodium acetate was not further increased with the further increase in ozone concentration from 37 mg/L to 50 mg/L, suggesting that the adsorption and reaction of ozone on the catalyst surface reach saturation. The removal of sodium acetate must be carried out by the absorption of ozone at the catalyst reaction site, and the further formation of other reactive oxygen species to attack the pollutants [24]. The result exhibited in Figure 7a suggests that sodium acetate was hard to eliminate in the absence of a catalyst as an end product of ozone oxidation itself.

The dosage of catalyst was also optimized at an ozone concentration of 50 mg/L, as shown in Figure 8b. The catalyst Mn_0.12_/CFA(A+B) at a concentration of 2.0 g/L had a sodium acetate removal efficiency of 40.5% in 60 min, much higher than that of Mn_0.12_/CFA(A+B), with concentration of 1.0 g/L. The increased dosage of catalysts partly provided more active sites to promote the production of active radicals from ozone decomposition and facilitate the degradation of sodium acetate [47]. As shown in Figure 8c, we also investigated the influence of different sodium acetate concentrations on the catalytic performance at the ozone concentration of 50 mg/L and the catalyst dosage of 2.0 g/L. It can be found that the ozone catalytic performance of Mn_0.12_/CFA(A+B) did not change significantly with the increase in sodium acetate concentration from 100 mg/L to 150 mg/L. However, when the concentration of sodium acetate was further increased to 200 mg/L or 250 mg/L, the ozone catalytic performance declined sharply. Excessive sodium acetate would compete with the dissolved ozone for limited surface active sites and ROS generation, further restricting the degradation of sodium acetate [48].

The pH of the solution is considered to be one of the key factors affecting the ozone catalytic process, which has a remarkable influence on the pathways of ozone reaction. According to the literature, ozone molecules are inclined to combine with organic matter with specific functional groups through selective reactions such as electrophilic, nucleophilic and dipolar addition in acidic solutions [49]. Thus, the products of the negatively charged ROS, such as ·OH and ·O^2−^, were inhibited when the pH < 7 [50]. Figure 8d shows the effect of solution pH on the ozone catalytic activity. As can be seen from the figure, the removal efficiency of sodium acetate was greatly affected when the pH values were set to 4.0 and 5.5. When the pH value was increased to 7.5 and 10.8, the removal efficiency of sodium acetate was also improved, which indicated that a less alkaline environment facilitated the transformation of ozone into those ROS, either in solution or on the catalyst’s surface. Besides this, the ozone catalytic performance of sodium acetate was hardly suppressed when the pH value was further increased to 12.0, suggesting the good applicability of the Mn-modified fly ash with a wide pH range.

### 3.4. ROS Generation over Mn-Modified Fly Ash

**Figure 9:**
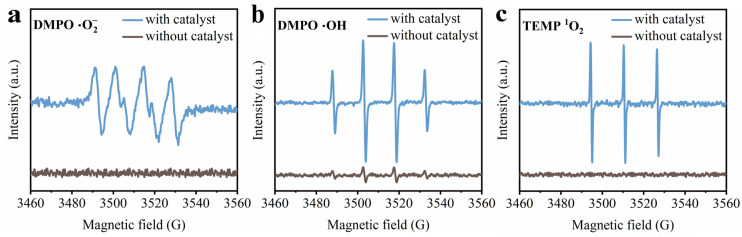
EPR spectra of (**a**) DMPO-·O^2−^, (**b**) DMPO-·OH and (**c**) TEMP-^1^O_2_.

The generation of ROS in the ozone catalytic process over catalyst Mn_0.12_/CFA(A+B) was confirmed through EPR measurements using 5,5-dimethyl-1-pyrroline N-oxide (DMPO) and 2,2,6,6-tetramethyl-4-piperidone hydrochloride (TEMP) as the capture agent. As shown in Figure 9a, typical peaks with an intensity ratio of 1:2:2:1 were observed under the ozone condition, which could be assigned to DMPO-·OH [51], indicating the presence of ·OH. The EPR signal with an intensity ratio of 1:1:1:1 in Figure 9b was ascribed to the adducts of DMPO-··O^2−^ combined with ·O^2-^ [52], and the EPR signal with an intensity ratio of 1:1:1 exhibited in Figure 9c represents the existence of 2,2,6,6-tetramethylpiperidine oxide (TEMPO), which suggests the existence of ^1^O_2_ [53]. The above results imply that ·O^2−^, ·OH and ^1^O_2_ were generated simultaneously via the ozone reaction over the catalyst Mn_0.12_/CFA(A+B).

**Figure 10:**
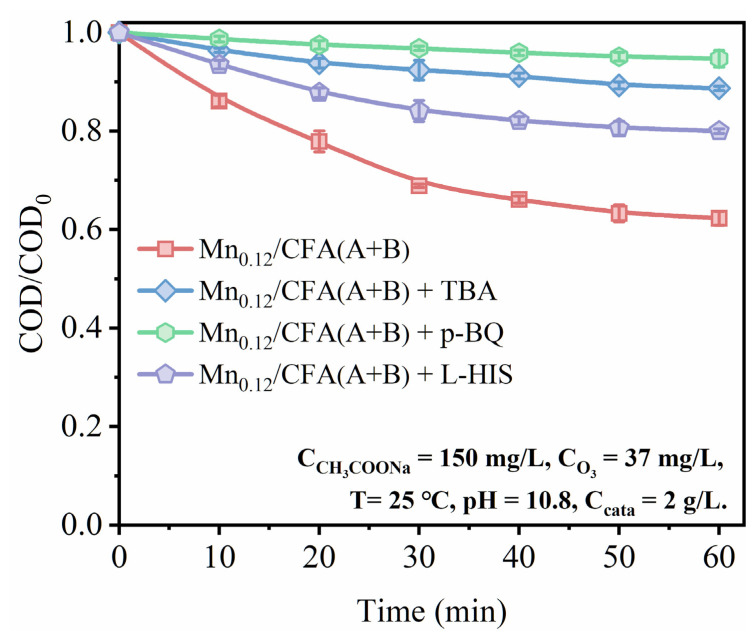
Removal rate of sodium acetate over catalyst Mn_0.12_/CFA(A+B) under the condition of different scavenger additions.

To further explore the influence of the related ROS on the ozone catalytic process over the catalyst Mn_0.12_/CFA(A+B), a variety of scavengers were also added into the reaction. Among them, p-benzoquinone (*p*-BQ) was used to quench ·O^2−^, tert-butyl alcohol (TBA) was used to scavenge ·OH and L-histidine (L-HIS) was used to scavenge singlet oxygen (^1^O_2_) [54]. We can infer from Figure 10 that the performance of the catalyst Mn_0.12_/CFA(A+B) deteriorated to varying degrees after three different scavengers were added individually, proving that ·OH, ·O^2−^ and ^1^O_2_ participated together in the oxidation elimination of sodium acetate. Besides this, it can also be deduced that ·O^2−^ was the most dominant oxidizing group by observing that the removal of sodium acetate was completely inhibited after the addition of *p*-BQ.

### 3.5. Reusability

**Figure 11:**
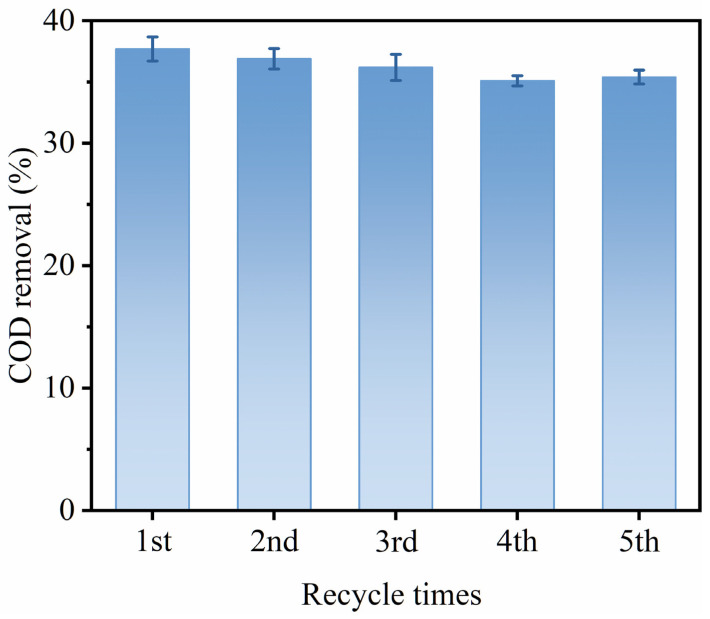
Cyclic performance test for degradation of sodium acetate.

The cyclic performance of catalysts is very important for the ozone catalyst. As shown in Figure 11, 150 mg/L sodium acetate was degraded over the Mn_0.12_/CFA(A+B) catalyst under the condition of ozone concentration = 37 mg/L, temperature = 25 °C, pH = 10.8 and catalyst amount = 2 g/L. The results show that the removal rates of sodium acetate in five cycles were basically the same, demonstrating that the Mn-modified fly ash catalyst had stable physical and chemical properties, making it suitable for recycling in the ozone catalysis process. This is mainly due to the irregular pore structure on the surface of alkali-treated fly ash, which is conducive to the removal of residual surface reactants in the circulation experiment. In addition, it can be seen from the results of the cycle experiment that the MnOx after calcination can be more tightly bonded to the surface of these pore structures. It will not fall off with the repeated treatment of the catalyst’s surface during the cycle experiment, ensuring the activation of the ozone.

## 4. Conclusions

In this work, a supported ozone catalyst was synthesized using fly ash as the carrier and manganese oxide as active phase. The structural properties of preprocessed fly ash samples were characterized by XRD, FESEM, TEM, ICP and N_2_ sorption tests. The S*_BET_* of the fly ash samples increased from 1.47 m^2^/g to 59.6 m^2^/g after being treated with acid and alkali processes. After being loaded with manganese oxide, the Mn_0.12_/CFA(A+B) catalyst exhibited strong ozone catalysis performance, which is attributed to the generation of ·OH, ·O^2−^ and ^1^O_2_ over oxygen vacancy with the existence of ozone. Sodium acetate was selected as the contaminant probe to evaluate the performance of this Mn-modified fly catalyst. The removal efficiency of sodium acetate over the sample of Mn_0.12_/CFA(A+B) can be maximized (150 mg/L) at ~40% under the conditions of ozone concentration = 50 mg/L, temperature = 25 °C, pH = 10.8 and catalyst amount = 2 g/L. The cyclic test results confirm the very good stability of the ozone catalyst synthesized by fly ash as a raw material. This work demonstrates that the industrial waste of fly ash can be re-synthesized as a stable and efficient ozone catalyst, which outlines a new research idea for wastewater treatment.

## Figures and Tables

**Table 1:** Porous parameters and element contents on different Mn-modified CFA samples from nitrogen sorption and ICP analysis.

Samples	S*_BET_* (m^2^/g)	V*_total_* (cm^3^/g)	Pore Size (nm)	ICP Analysis (10^−2^ g/g)				
				Si	Al	Ca	Fe	Mg
CFA	1.47 ± 0.02	0.0047	8.14	19.04	16.03	3.99	3.77	0.74
CFA(A)	11.37 ± 0.26	0.0083	9.72	15.71	15.15	1.03	1.32	0.21
CFA(A+B)	59.64 ± 0.81	0.13	12.761	10.83	13.73	0.67	0.98	0.13

**Table 2:** Relative contents of surface elements on different Mn-modified CFA samples from XPS analysis.

Samples	ICP Analysis	XPS Analysis			
	Mn wt.%	Mn^3+^/Mn^4+^	O_ads_	O_sur_	O_lat_
CFA(A+B)	/	/	0.56	0.39	0.05
Mn_0.04/_CFA(A+B)	3.61%	0.75	0.46	0.43	0.11
Mn_0.08_/CFA(A+B)	6.97%	1.23	0.40	0.48	0.12
Mn_0.12_/CFA(A+B)	11.10%	0.93	0.32	0.50	0.18

## Data Availability

Not applicable.

## Supplementary Materials

The following supporting information can be downloaded at: https://www.mdpi.com/article/10.3390/toxics11080700/s1, Figure S1. SEM images of (A1–A2) Mn_0.08_/CFA, (B1–B2) Mn_0.08_/CFA(A), and (C1-C2) Mn_0.08_/CFA(A+B); Figure S2. The degradation performance of sodium acetate over catalysts Mn_0.08_/CFA(A+B), Mn_0.08_/CFA(A), and Mn_0.08_/CFA (reaction condition: ozone concentration = 37 mg/L, temperature = 25 °C, pH = 10.8 and catalyst amount = 2 g/L).
