# Supplementary material for: Degradation of Sodium Acetate by Catalytic Ozonation Coupled with a Mn-Functionalized Fly Ash: Reaction Parameters and Mechanism
Source: Toxics. 2023 Aug 14;11(8):700. doi: 10.3390/toxics11080700 (PMC10457793; doi:10.3390/toxics11080700)
Supplement: Supplementary file 1 [file toxics-11-00700-s001.zip › toxics-2469708-supplementary.pdf]

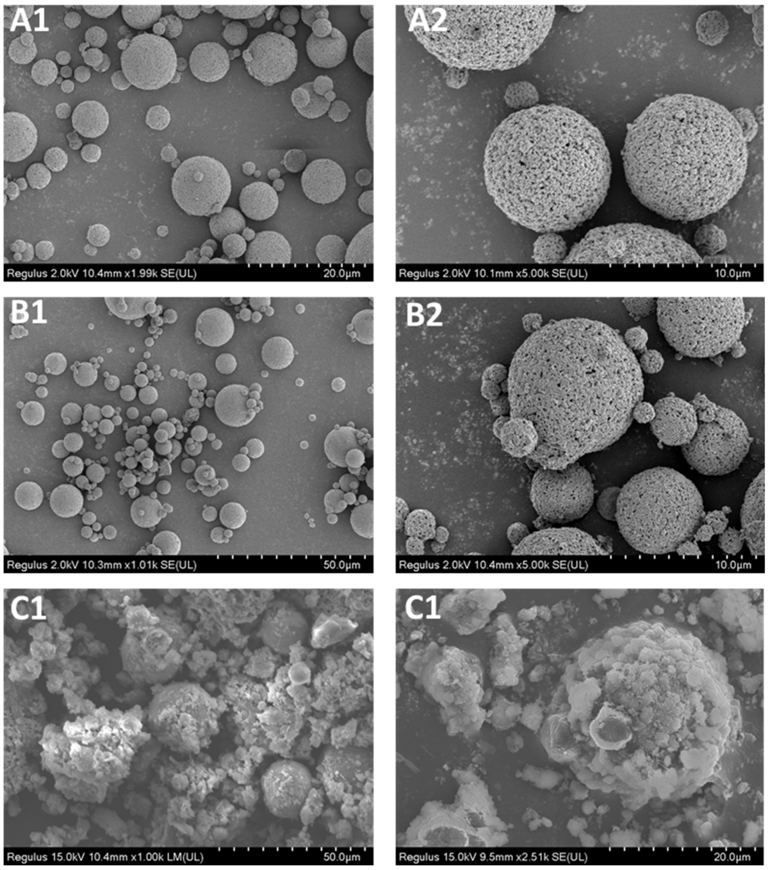

Figure S1. SEM images of (A1-A2) $Mn_{0.08}$/CFA, (B1-B2) $Mn_{0.08}$/CFA(A), and (C1-C2) $Mn_{0.08}$/CFA(A+B).

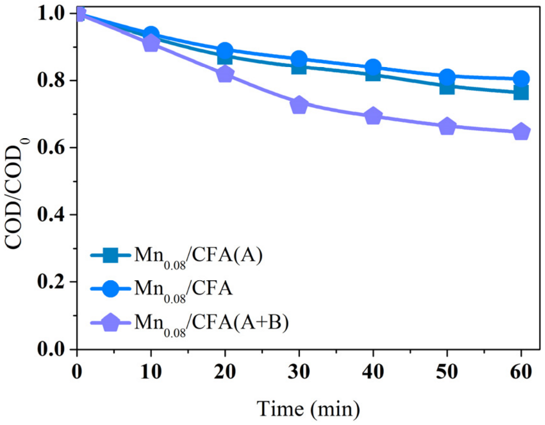

Figure S2. The degradation performance of sodium acetate over catalysts $Mn_{0.08}$/CFA(A+B), $Mn_{0.08}$/CFA(A), and $Mn_{0.08}$/CFA (reaction condition: ozone concentration = 37 mg/L, temperature = 25 °C, pH = 10.8 and catalyst amount = 2 g/L).
